# 

**Published:** 1863-05

**Authors:** 


### DENTAL REGISTER

### OF THE WEST.

EDITED BY

J. TAFT AND GEO. WATT.

MAY,—1863.

**MONTHLY:**

**Three Dollars per Annum, in advance.**

CINCINNATI:

J. TAFT, PUBLISHER.

J. TAFT, DENTIST, 172 RACE STREET.

